# Pharmacokinetics, Immunogenicity and Safety Study for SHR-1309 Injection and Perjeta® in Healthy Chinese Male Volunteers

**DOI:** 10.3389/fphar.2021.660541

**Published:** 2021-06-02

**Authors:** Yingzi Cui, Dongyang Cui, Xinran Ren, Xuesong Chen, Guangwen Liu, Zhengzhi Liu, Yanli Wang, Xinyao Qu, Yicheng Zhao, Haimiao Yang

**Affiliations:** ^1^ Phase I Clinical Trial Laboratory, Affiliated Hospital of Changchun University of Chinese Medicine, Jilin, China; ^2^ Jiangsu Hengrui Medicine Co.,Ltd., Jiangsu, China; ^3^ Shanghai Hengrui Pharmaceutical Co., Ltd., Shanghai, China; ^4^ School of Pharmacy, Jilin University, Jilin, China; ^5^ Clinical Medical College, Changchun University of Chinese Medicine, Jilin, China; ^6^ Jilin Province Honesty Medical Technology Consulting Co., Ltd., Jilin, China

**Keywords:** perjeta®, pharmacokinetics, bioequivalence, immunogenicity, safety, SHR-1309 injection

## Abstract

**Objectives:** Pertuzumab is a monoclonal antibody for the treatment of breast cancer. The aim of this study was to compare the pharmacokinetics, immunogenicity and safety of the test preparation SHR-1309 injecta and the reference preparation Perjeta® in healthy Chinese male subjects.

**Methods:** In this randomized, double-blind, single dose, two-way, parallel bioequivalence trial, a total of 80 qualified Chinese male subjects were selected and randomly divided into two groups. Each subject was intravenously injected with SHR-1309 or Perjeta®. Blood samples were collected at 21 different time points for pharmacokinetic analysis. In addition, immunogenicity was assessed at five different time points. The safety of the medication was monitored throughout the whole trial.

**Results:** C_max_ and AUC_0-t_ were the primary pharmacokinetic parameters. Under a 90% confidence interval, their geometric mean ratios were 98.30 and 88.41% for SHR-1309 injection and Perjeta®, respectively. The geometric mean ratio of secondary pharmacokinetic parameters AUC_0-∞_ was 88.58%. These evaluation indexes are in the standard range of 80–125%, so SHR-1309 can be considered bioequivalent to Perjeta®. After 1,680 h (day 70) of administration, the two groups had 12 and 13 subjects who produced antidrug antibody (ADA), respectively. The occurrence time and proportion of ADA in SHR-1309 and Perjeta® were similar between subjects, and they had similar immunogenicity. During the entire trial period, there were 71 drug-related adverse reactions in 29 subjects who received SHR-1309 and 61 drug-related adverse reactions in 32 subjects who received Perjeta®. The incidence of adverse reactions between the two drugs was similar.

**Conclusion:** The pharmacokinetic parameters, immunogenicity and safety of the biosimilar SHR-1309 injection produced by Shanghai Hengrui Pharmaceutical Co. Ltd. were similar to the original drug Perjeta® produced by Roche Pharma AG. The two drugs met the bioequivalence evaluation criteria. Therefore, SHR-1309 is bioequivalent to Perjeta®. Clinical trial registration: CTR20200,738.

## Introduction

Breast cancer (BC) is one of the most common malignant tumors in women (Giri et al., 2018; Li et al., 2018; Tsoutsou et al., 2018). In China, the incidence rate of female breast cancer is increasing each year, and the incidence is second only to cervical cancer (Yu et al., 2017). Breast cancer can be divided into four subtypes: luminal A, luminal B, human epidermal growth factor receptor two (HER2)-overexpressing breast cancer and triple-negative breast carcinoma (Yu et al., 2017; Chan et al., 2021). Around 25–30% of breast cancer patients exhibit overexpression of HER2 (Yu et al., 2017). In China, 27–49% of breast cancer patients are HER2-positive (Wang et al., 2020). HER2-overexpressing breast cancer has a poor prognosis and often relapses (Swain et al., 2015; Wang et al., 2020), representing an important public health problem in many countries. Currently, HER2 is recognized as one of the most common targets in breast cancer treatment (Yu et al., 2017). With the in-depth study of tumor mechanisms, new targeted drugs, especially immunotherapies, have become a research hotspot for the treatment of tumors.

HER2 belongs to the human epidermal growth factor receptor (EGFR) family. It is a biomarker expressed on the cancer cell membrane and is overexpressed in HER2-positive breast cancer (Osako et al., 2015). HER2 binds with ligands to form dimers, and its tyrosine residues become phosphorylated, resulting in activation of mitogen-activated protein kinase (MAPK) and phosphoinositide-3-kinase-protein kinase B/Akt (PI3K-PKB/Akt) signal transduction pathways. Thus, HER2 can promote cell growth and differentiation and stimulate proliferation, invasion and antiapoptotic ability in cancer cells (Piccart-Gebhart et al., 2005; Paek et al., 2019). Therefore, HER2 is an effective target for inhibiting HER2-positive breast cancer.

Currently, the drugs used to target HER2 for the treatment of breast cancer primarily include trastuzumab, lapatinib, Pertuzumab and ado-trastuzumab emtansine (Figueroa-Magalhaes et al., 2014; Bartsch and Bergen, 2018). Trastuzumab was the first anti-HER2 antibody and has a good therapeutic effect on HER2-positive breast cancer (Osako et al., 2015; Li et al., 2018; Zhu et al., 2020). Pertuzumab (trade name: Perjeta®) is another HER2 monoclonal antibody that blocks formation of the HER2 dimer and its receptor by binding HER2, inhibiting activation of the PI3K-PKB/Akt signaling pathway, inhibiting the activity of cancer cells, slowing tumor growth, and reducing recurrence rate (Osako et al., 2015; Williams et al., 2017; Paek et al., 2019). In addition, because Pertuzumab and trastuzumab work on different domains of the HER2 protein, the combination of Pertuzumab and trastuzumab has a synergistic effect on cancer cells (Lam et al., 2015; Osako et al., 2015; Swain et al., 2015; Williams et al., 2017). Perjeta® was approved by the FDA on June 8, 2012. It is used in combination with trastuzumab and docetaxel in the treatment of HER2-positive metastatic breast cancer patients who have not previously received anti-HER2 therapy or chemotherapy, as well as neoadjuvant therapy for early breast cancer. In December 2018, Perjeta® was approved for use in China.

Biosimilar refers to biological products containing the approved active substances of the original biological drugs (Zhang et al., 2020a; Zhang et al., 2020b). The emergence of biosimilars can provide more advanced treatment options for larger numbers of patients (Bushra et al., 2020; Farahani et al., 2020). At the same time, it can also reduce the cost of health care (Zhang et al., 2020a; Farahani et al., 2020). The production process of biological agents is complex, and any slight difference may cause the final product to fail (Zhang et al., 2020a; Zhang et al., 2020b). Therefore, biosimilars should be comparable with reference preparations in terms of quality, biological activity, safety and efficacy to ensure their safety and effectiveness before they can be approved for marketing (Zhang et al., 2020a; Farahani et al., 2020; Finck et al., 2020; Zhu et al., 2020). It is very important to study the pharmacokinetics (PK), efficacy, safety and immunogenicity of biosimilars (Shin et al., 2020).

SHR-1309 is a biological product developed by Shanghai Hengrui Pharmaceutical Co., Ltd. SHR-1309 is consistent with Perjeta®’s structure and glycosylation and has high similarity with respect to various pharmacological, pharmacodynamic and pharmacokinetic parameters *in vitro* and *in vivo*. The aim of this study was to compare SHR-1309 with Perjeta® in healthy Chinese male volunteers through a randomized, double-blind, single dose, two-way, parallel trial, and their bioequivalence, immunogenicity and safety were evaluated.

## Methods

### Subjects

First, subjects who met the inclusion criteria were selected for this phase I clinical trial. Before screening, participants were given a full description of the nature, purpose, content, procedure and any adverse reactions that might occur. A total of 175 male subjects were selected. Based on the inclusion and exclusion criteria, 93 potential subjects were excluded, 82 were selected, and two were candidates. All subjects voluntarily signed the informed consent form approved by the ethics committee. This study conforms to the ethical principles of Declaration of Helsinki, each subject was fully respected during the trial. Subjects were aged 18–55 years and weighed no less than 50 kg. Body mass index (BMI) was within 19–26. The ethics committee of the Affiliated Hospital to Changchun University of Chinese Medicine approved this study protocol (No. CCZYFYLL 2018-101-1). This trial has been registered in the Chinese Clinical Trial Registry (ChiCTR) (No. CTR20200,738).

### Design

This study used a single center, randomized, double-blind, single dose, parallel controlled design. Eighty subjects were randomly divided into two groups, and each group had 40 cases. They were given SHR-1309 (specification: 14 ml/0.42 g, manufacturer: Shanghai Hengrui Pharmaceutical Co., Ltd., batch number: P1805) or Perjeta® (specification: 14 ml/0.42 g, manufacturer: Roche Pharma AG, batch number: H0295H05) with a single intravenous infusion of 3 mg/kg. Venous blood was collected at 21 time points for blood concentration detection, including 0 h (before administration), 30 min, 60 min, 1.5, 3, 6, 12, and 24 h (day 1), 48 h (day 2), 72 h (day 3), 96 h (day 4), 168 h (day 7), 240 h (day 10), 336 h (day 14), 504 h (day 21), 672 h (day 28), 840 h (day 35), 1,008 h (day 42), 1,344 h (day 56), 1,680 h (day 70), and 2016 h (day 84). Venous blood was also collected at 0 h, 336 h (day 14), 1,008 h (day 42), 1,680 h (day 70) and 2016 h (day 84) for the immunogenicity test. The volume of venous blood collected at each time point was approximately 4 ml. After 30 min of coagulation at room temperature, serum samples were centrifuged at 4°C and 1,300 g for 10 min for pharmacokinetic and immunogenicity studies. Blood samples were collected from ADA-positive subjects at 7 months (±21 days), 13 months (±21 days), 19 months (±21 days) and 24 months (±21 days) for immunogenicity tests. ADA-positive subjects were followed up until they turned negative or until 24 months after administration.

### Quantification of Serum Pertuzumab Concentrations and Immunogenicity Assessment

Plasma concentration was determined by enzyme-linked immunosorbent assay (ELISA. A total of 1672 PK samples were detected. The anti-SHR-1309 antibody (purchased from GenScript) was coated with 96 microplates. SuperBlock Blocking Buffer (purchased from Thermo Scientific, Cat No. 37515) was added to block the nonspecific binding site. The standard, quality control samples and test samples were diluted 1:100 and then added to 96-well microplates. SHR-1309 was specifically captured by the precoated antibody on the microplate. After incubation and washing, biotin-labeled antibody (purchased from Covance in house) was added and incubated. After washing, streptavidin-HRP (purchased from Immunoresearch, Cat No. 016-030-084) was added for incubation. After the final washing, TMB (purchased from KPL, Cat No. 52-00-02) substrate solution was added to the microplate. The reaction products were detected by spectrophotometry. The plasma drug concentration was calculated using a standard regression curve.

The ADAs of SHR-1309 and Perjeta® in the serum were detected by electrochemiluminescence (ECL). A total of 398 ADA samples were detected. First, all samples underwent preliminary screening analysis, and samples whose initial screening analysis value was higher than or equal to the screening critical value were considered suspected positive samples. Suspected positive samples proceeded into specificity analysis, which was similar to the screening analysis, but the samples were detected simultaneously with or without drug. The sample with true-positive antibody produced a significant inhibitory response after administration.

### Pharmacokinetics Assessments

The maximum observed drug concentration in the plasma (C_max_) and the AUC of the analyte in the plasma over the time interval from time zero to the last measurable concentration (AUC_0-t_) are used as the main parameters to evaluate the PK similarity between the test and reference preparation. The area under the curve from 0 to infinity (AUC_0-∞_) is used as a secondary evaluation parameter. In the 90% confidence interval (CI), if the ratio of the geometric mean of SHR-1309 to Perjeta® is within the range of 80–125%, it can be considered that the two compounds are bioequivalent. Other pharmacokinetic parameters are used as supplementary evaluation parameters, including the time from administration to the maximum observed concentration of the analyte in the plasma (T_max_), the terminal half-life of the analyte in the plasma (t_1/2z_), the steady-state apparent distribution volume measured after intravenous administration (V_ss_), distribution volume (V_z_), clearance rate (CL_z_), terminal rate constant in the plasma (λ_z_), mean residence time from zero to the lowest detectable concentration (MRT_0-t_), mean residence time extrapolated from zero to infinity (MRT_0-∞_), and AUC_%Extrap_ ([(AUC_0-∞_-AUC_0-t_)/AUC_0-∞_] × 100%).

### Safety

The vital signs of subjects were monitored before and after administration, including electrocardiogram, blood pressure, heart rate, blood oxygen saturation, temperature, pulse, etc. After 4 days, subjects were allowed to leave the clinical research center, and they needed to return for safety examinations at different time points. In accordance with the definition of adverse events in Good Clinical Practice of Pharmaceutical Products (GCP), AEs were recorded after administration. From the beginning of administration to the end of follow-up, any adverse events reported by subjects or observed by researchers that should be recorded truthfully, including occurrence time, severity, duration, measures and outcome. The severity of adverse events was judged by referring to the Common Terminology Criteria Adverse Events (CTCAE 5.0) and the judgment of clinicians.

### Sample Size and Statistical Methods

According to previous reports regarding the variation coefficient of HER-2 inhibitor trastuzumab comparison and other biosimilar drug trials (Wisman et al., 2014; Knight et al., 2016). It is estimated that the variation coefficient of the main pharmacokinetic parameters of Patuzumab in healthy people is about 25%. When α = 0.05, the confidence interval of equivalence was 80.00–125.00%, the expected θ value was between 0.95 and 1.05, and the degree of assurance was 0.85 (β = 0.15). The parallel design estimation was performed *via* PASS 11.0.7, and the result indicated that there were two groups which contains at least 31 subjects were required for this trial. Considering the possible withdrawal and drop out of subjects, the number of samples in each group was set to 40, and a total of 80 subjects were enrolled.

WinNonlin 8.1 was employed to perform noncompartmental model fitting. The main pharmacokinetic parameters of the subjects were calculated, including C_max_, AUC_0-t_, AUC_0-∞_, CL_z_, t_1/2z_, V_z_, V_ss_, MRT_0-t_, and MRT_0-∞_. These parameters fully reflect the characteristics of drug distribution and elimination in the human body. After logarithmic transformation, analysis of variance (ANOVA) was used to analyze the data. Under 90% CI, the geometric mean ratio of the main parameters was calculated. We determined whether the ratio was within 80.00–125.00% and then evaluated the pharmacokinetic similarity between SHR-1309 and Perjeta®. T_max_ was analyzed by rank sum test. The occurrence time and incidence of ADA positivity were statistically analyzed. The incidences of adverse events and serious adverse events during the trial were recorded.

## Results

### Subjects

**FIGURE 1:**
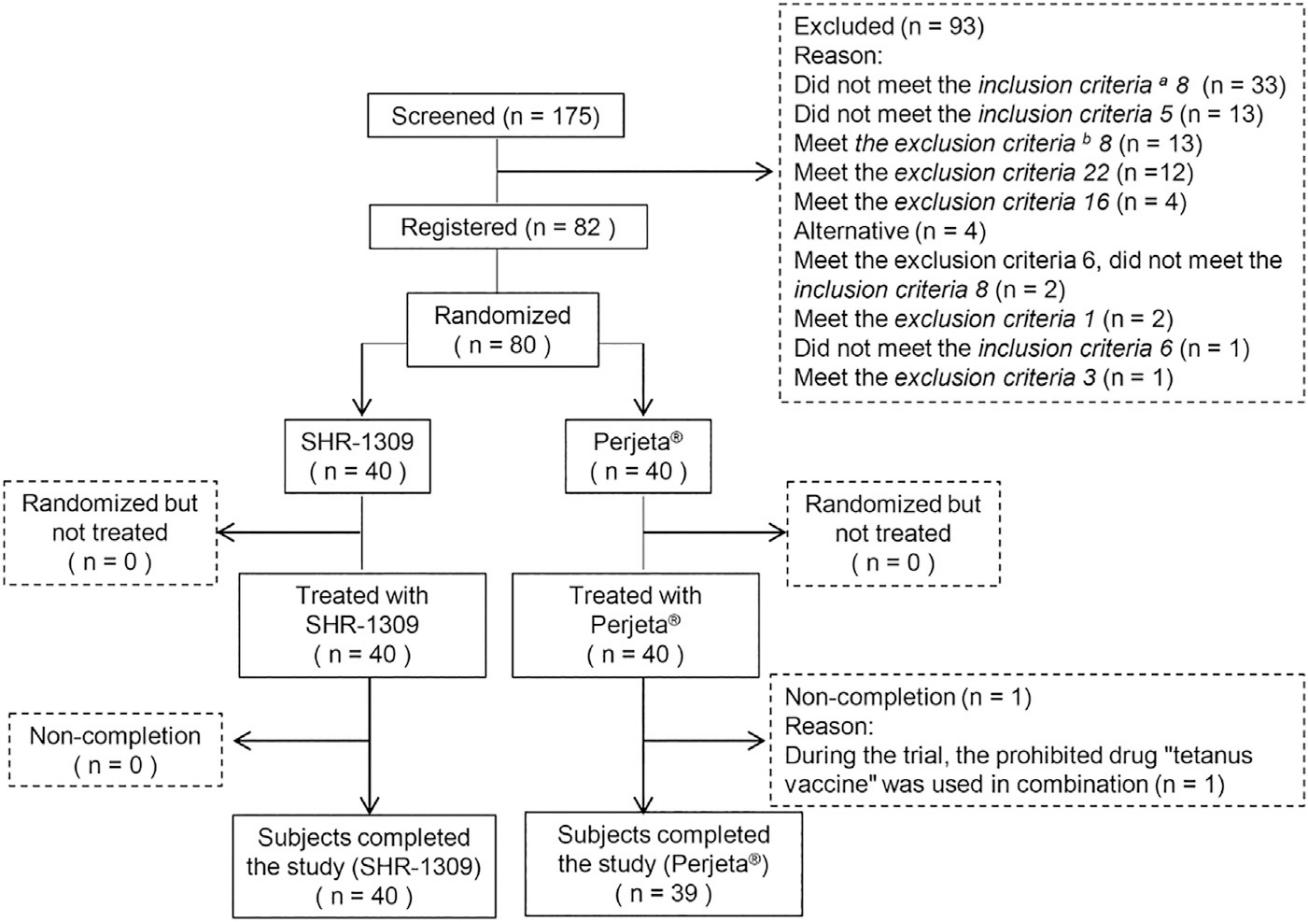
Subject flow chart. *n* indicates the number of subjects. Exclusion indicates subjects who did not meet the inclusion criteria or who met the exclusion criteria. Replacement indicates that the subjects did not meet the criteria before drug administration. Non-completion indicates that the subjects did not complete all sample collections for some reason and were not included in the final statistical analysis. a,b: the detailed inclusion criteria and exclusion criteria are shown in the supplementary file.

According to the inclusion and exclusion criteria (supplemental file), 82 subjects were selected from 175 male subjects. The number of final subjects was 80 due to the withdrawal of two subjects before the trial. Participants were randomly divided into two groups, with 40 in each group. They were given intravenous administration of SHR-1309 or Perjeta®. Except for one subject in the Perjeta® group who did not complete the study, all other subjects completed the trial (Figure 1). The average age of the SHR-1309 group was 39.8 years old, ranging from 21 to 55 years old, the body weight was 52.9–85.5 kg, and the BMI was 19.4–26. The average age of the Perjeta® group was 38.2 years old, ranging from 24 to 55 years old, the body weight was 53.5–82.4 kg, and the BMI was 19.9–26. The basic parameters of the two groups were similar. Subject details are shown in Table 1.

**TABLE 1:** Subject demographics.

	SHR-1309 injection (*N* = 40)	Perjeta® (*N* = 40)
Age (years)	39.8 ± 10.89	38.2 ± 8.97
Mean ± SD	28.5–48.0	32.0–45.5
Q1-Q3	21–55	24–55
Min-Max	42.5	37.5
Median		
Age stratification, n (%)	19 (47.5)	23 (57.5)
18–40	5 (12.5)	7 (17.5)
41–45	16 (40.0)	10 (25.0)
46–55		
Nation, n (%)	35 (87.5)	38 (95.0)
Ethnic han	5 (12.5)	2 (5.0)
Other nationalities		
Occupation, n (%)	14 (35.0)	11 (27.5)
Manual labor	26 (65.0)	29 (72.5)
Non-manual work		
Marriage, n (%)	8 (20.0)	10 (25.0)
Unmarried	26 (65.0)	26 (65.0)
Married	5 (12.5)	4 (10.0)
Divorced	1 (2.5)	0 (0)
Widowed		
Height (cm)	169.96 ± 6.272	170.83 ± 5.488
Mean ± SD	165.25–174.75	167.00–174.00
Q1-Q3	156–182	161–182
Min-max	170.25	170.25
Median		
Weight (kg)	65.58 ± 7.740	66.43 ± 7.384
Mean ± SD	60.35–72.05	59.55–72.20
Q1-Q3	52.9–85.5	53.5–82.4
Min-max	64.00	67.50
Median		
Weight stratification n (%)	9 (22.5)	10 (25.0)
50–60 kg	17 (42.5)	15 (37.5)
60–70 kg	13 (32.5)	14 (35.0)
70–80 kg above 80 kg	1 (2.5)	1 (2.5)
BMI (kg/m^2^)	23.04 ± 2.125	23.17 ± 1.830
Mean ± SD	21.10–24.80	21.95–24.65
Q1-Q3	19.4–26	19.9–26
Min-max	23.40	23.30
Median	—	—

SD: Standard deviation; Q1: first quartile; Q3: third quartile; Min: minimum; Max: maximum; BMI: body mass index.

### Pharmacokinetics

**FIGURE 2:**
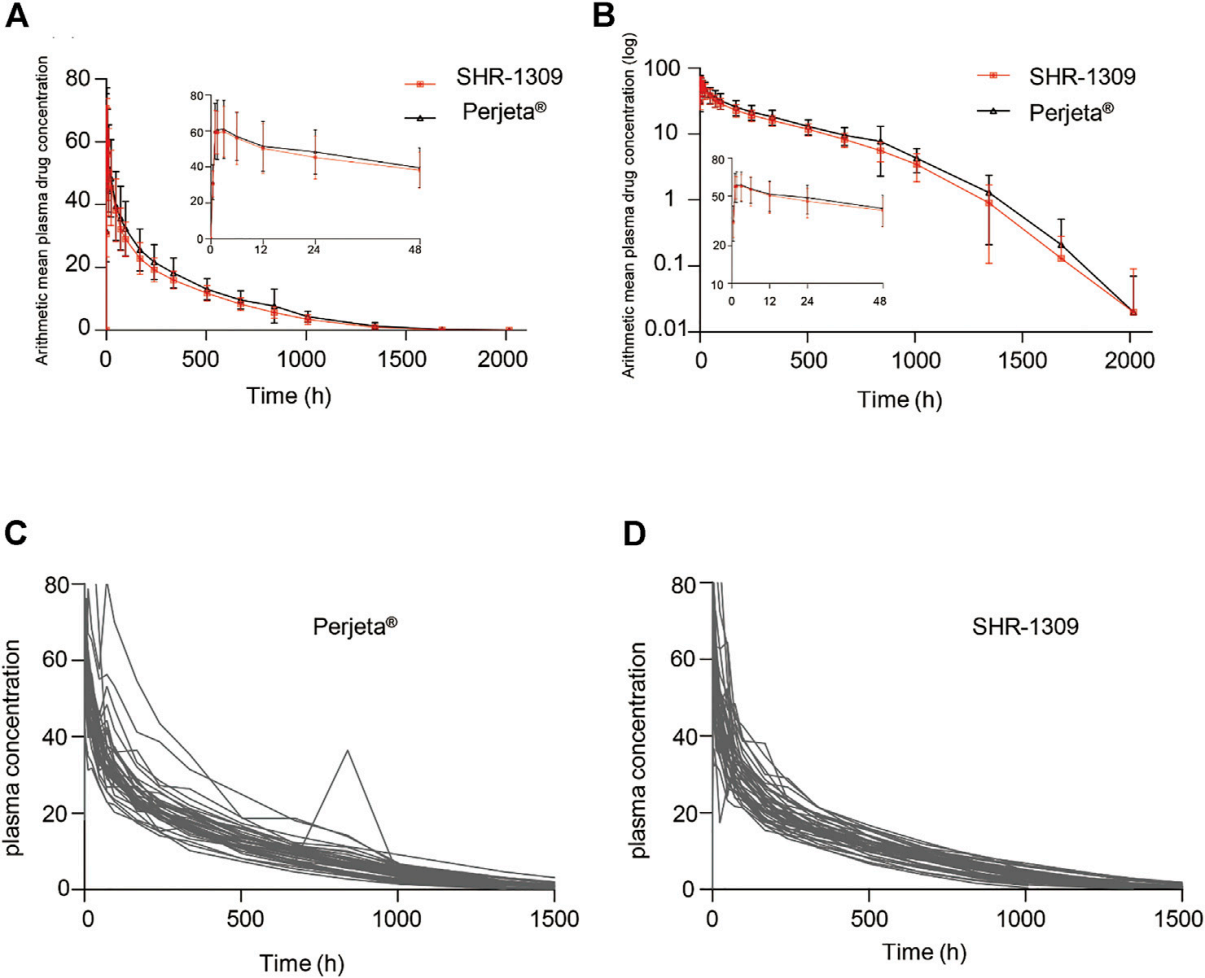
Mean blood concentration time curve. **(A)** Mean plasma concentration ( ±SD) time curve after intravenous drip of SHR1309 or Perjeta®. **(B)** Logarithmic transformation of the mean plasma concentration ( ±SD) time curve after intravenous dripping of SHR1309 or Perjeta®. **(C)** Plasma drug concentration time curve of 39 subjects after intravenous drip of Perjeta®. **(D)** Plasma drug concentration time curve of 40 subjects after intravenous drip of SHR1309.

**FIGURE 3:**
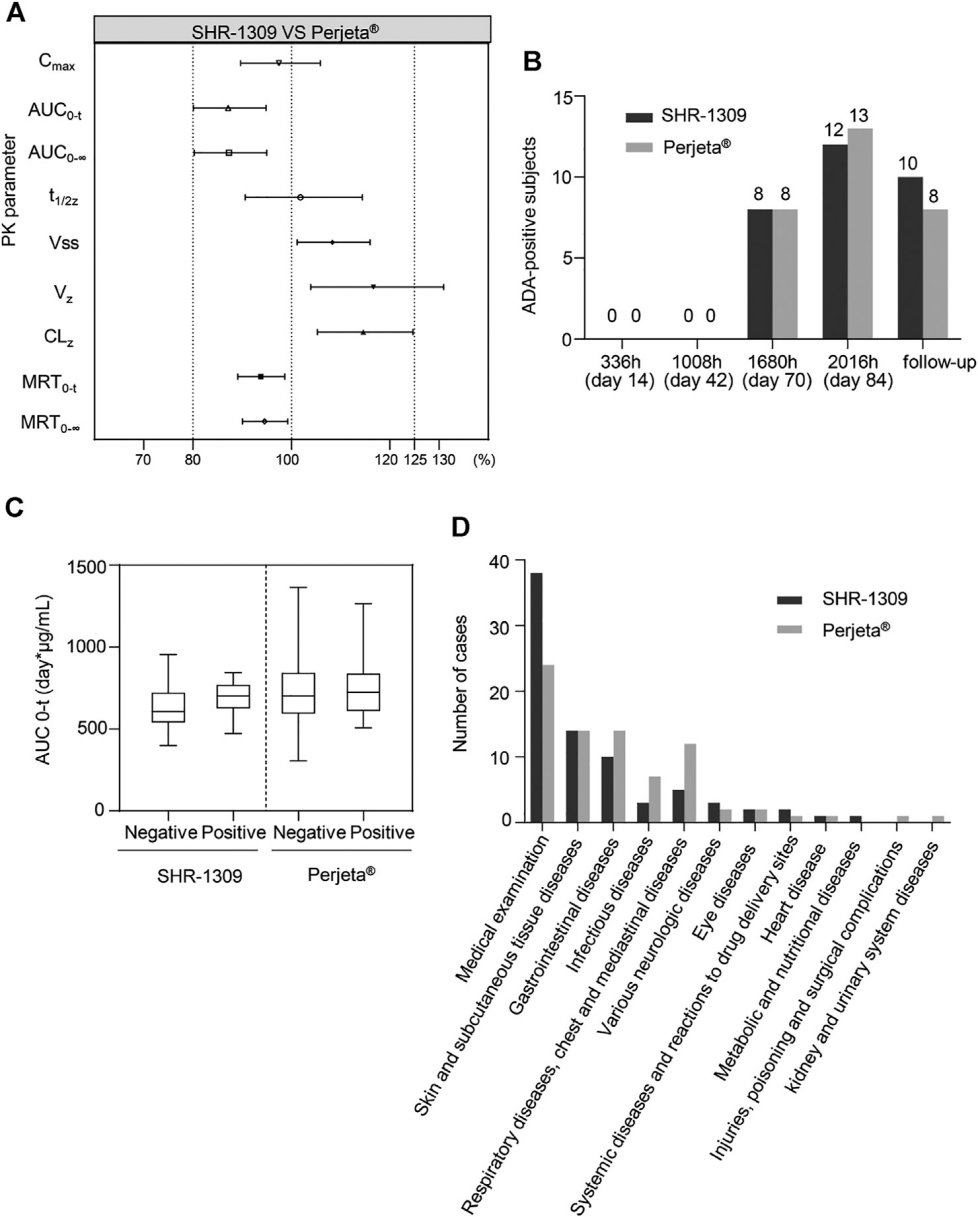
Bioequivalence, immunogenicity and safety analysis of SHR1309 and Perjeta®. **(A)** Under 90% CI, the geometric ratio ranges of PK parameters for SHR1309 and Perjeta®. PK: pharmacokinetic. **(B)** After intravenous drip of SHR1309 or Perjeta®, the number of ADA-positive subjects at four different time points (336, 1008, 1680, and 2016 h) and at follow-up time was analyzed. **(C)** Comparison of AUC values for ADA-positive and -negative subjects in SHR1309 and Perjeta®, respectively. ADA: antidrug antibody. AUC_0-t_: the AUC of the analyte in the plasma over the time interval from time zero to the last measurable concentration. The solid line in the middle represents the median. **(D)** The TEAE of the two groups was categorized according to system organ, and the corresponding cases were quantified. TEAE: treatment-emergent adverse event.

To evaluate the bioequivalence of SHR-1309 and Perjeta®, we performed PK analysis on two groups of subjects. The subjects were sampled at 21 time points before and after drug administration. Plasma drug concentration was detected by ELISA, and the data were fitted to form the average plasma drug concentration curve of SHR-1309 and Perjeta® (Figure 2A). The logarithmic transformation of the curve is shown in Figure 2B. At the same time, the plasma drug concentration of each subject in the two groups was fitted (Figures 2C,D). There was no significant difference in blood concentration between the two groups after administration. The primary evaluation parameters, secondary evaluation parameters and other pharmacokinetic parameters were obtained through calculation of plasma drug concentration (Table 2; Supplementary Table S1). The mean and standard deviation (SD) values of Cmax were 63.40 ± 15.18 μg/ml and 64.58 ± 17.17 μg/ml for SHR-1309 and Perjeta®, respectively, and the ratio of the geometric mean was 98.30%. The mean and SD values of AUC_0-t_ were 653.37 ± 133.65 and 746.26 ± 197.06 day*μg/mL, respectively, and the ratio of the geometric mean was 88.41%. The mean and SD values of AUC_0-∞_ were 657.29 ± 133.29 and 749.70 ± 198.23 day*μg/mL, respectively, and the ratio of the geometric mean was 88.58%. Tmax was 1.50 and 3.00 h, respectively. The geometric mean values and ratios of all parameters are shown in Table 3. The primary pharmacokinetic parameters of SHR-1309 and Perjeta® were all up to the standard. Except for Vz, the 90% CI for all values fell within the 80%–125% range (Figure 3A). The PK parameter values of the two drugs were similar, and according to the PK evaluation standard of bioequivalence, SHR-1309 and Perjeta® are bioequivalent.

**TABLE 2:** The main PK parameters of SHR-1309 injection or Perjeta® after intravenous drip.

	SHR-1309 injection (*N* = 40)	Perjeta^®^ (*N* = 39)
C_max_ (μg/ml)	63.40 ± 15.18	64.58 ± 17.17
AUC_0-t_ (day*μg/mL)	653.37 ± 133.65	746.26 ± 197.06
AUC_0-∞_ (day*μg/mL)	657.29 ± 133.29	749.70 ± 198.23
T_max_ (h)	3.00 (0.99–48)	1.50 (1–72)
t_1/2z_ (day)	7.29 ± 2.42	7.06 ± 2.11
V_ss_ (ml/kg)	70.92 ± 11.91	66.04 ± 11.24
V_z_ (ml/kg)	49.26 ± 16.82	41.69 ± 10.94
CL_z_ (ml/h/kg)	0.20 ± 0.04	0.18 ± 0.04
λ_z_ (1/day)	0.11 ± 0.03	0.11 ± 0.03
MRT_0-t_ (day)	14.78 ± 2.08	15.61 ± 1.88
MRT_0-∞_(day)	15.12 ± 2.01	15.87 ± 1.93
AUC_%Extrap_ (%)	0.64 ± 0.76	0.45 ± 0.46

Mean ± SD was used to describe the parameters; T_max_ was described by median (min max); C_max_: the maximum observed drug concentration in the plasma; AUC_0-t_: the AUC of the analyte in the plasma over the time interval from time zero to the last measurable concentration; AUC_0-∞_: the area under the curve from 0 to infinity; T_max_: the time from administration to the maximum observed concentration of the analyte in the plasma; t_1/2z_: the terminal half-life of the analyte in the plasma; V_ss_: the steady-state apparent distribution volume was measured after intravenous administration; V_z_: distribution volume; CL_z_: clearance rate; λ_z_: terminal rate constant in the plasma; MRT_0-t_: mean residence time from zero to the lowest detectable concentration; MRT_0-∞_: mean residence time extrapolated from zero to infinity; AUC_%Extrap_ = [(AUC_0-∞_-AUC_0-t_)/AUC_0-∞_ × 100].

**TABLE 3:** Results of the equivalence determination of the test drug and reference drug.

PK parameter	Geometric mean		Comparison	
	SHR-1309 injection (*N* = 40)	Perjeta® (*N* = 39)	Ratio%	90%CI (%)
C_max_ (μg/ml)	61.92	63.53	97.47	89.66–105.9
AUC_0-t_ (day*μg/mL)	639.65	734.07	87.14	80.07–94.83
AUC_0-∞_ (day*μg/mL)	643.88	737.43	87.31	80.27–94.98
t_1/2z_ (day)	6.97	6.84	101.81	90.62–114.39
V_ss_ (ml/kg)	69.83	64.47	108.33	101.19–115.96
V_z_ (ml/kg)	46.86	40.17	116.67	103.93–130.96
CL_z_ (ml/h/kg)	0.19	0.17	114.59	105.30–124.69
MRT_0-t_ (day)	14.61	15.58	93.77	89.13–98.65
MRT_0-∞_(day)	14.61	15.84	94.54	90.04–99.26

PK: pharmacokinetic; CI: confidence interval; C_max_: maximum observed drug concentration in the plasma; AUC_0-t_: the AUC of the analyte in the plasma over the time interval from time zero to the last measurable concentration; AUC_0-∞_: the area under the curve from 0 to infinity; t_1/2z_: the terminal half-life of the analyte in the plasma; V_ss_: steady-state apparent distribution volume was measured after intravenous administration; V_z_: distribution volume; CL_z_: clearance rate; MRT_0-t_: mean residence time from zero to the lowest detectable concentration; MRT_0-∞_: mean residence time extrapolated from zero to infinity.

### Immunogenicity

ADA detection of antibody drugs is also an important index to evaluate bioequivalence. In this trial, 80 subjects were given an intravenous drip of antibody drugs. Each group had 40 participants. All subjects were ADA negative before administration. After administration, 12 cases (30.0%) in the Perjeta® group and 13 cases (32.5%) in the SHR-1309 group were ADA positive. After administration, the occurrence time for ADA positivity was 1,680 h (70 days) in both groups. There was no significant difference in the incidence or time of ADA positivity between SHR-1309 and Perjeta® (Table 4). The positive rates of ADA positivity for SHR1309 and Perjeta® were all increased in a time-dependent manner (Figure 3B). The presence of ADA did not affect AUC_0-t_ values of the two groups (Figure 3C). Therefore, we can conclude that SHR1309 and Perjeta® have similar immunogenicity.

**TABLE 4:** Summary of the incidence of ADA.

Time point	SHR-1309		Perjeta®	
	N	*n* (%)	*N*	*n* (%)
336 h	40	0 (0)	40	0 (0)
1,008 h	40	0 (0)	40	0 (0)
1,680 h	39	8 (20.0)	40	8 (20.0)
2016 h	39	12 (30.0)	40	13 (32.5)
Follow-up visit	40	10 (25.0)	40	8 (20.0)

*N* represents the total number of participants in the experiment, *n* is the number of ADA-positive participants, and % is the percentage of ADA-positive participants.

### Safety

Safety is very important for biosimilars. A total of 80 subjects were included in this safety evaluation. The results showed that 158 treatment-emergent adverse events (TEAEs) occurred in 62 subjects, and the incidence of adverse events was 77.5% (62/80). In the SHR1309 group, 79 TEAEs occurred in 29 subjects, and the incidence of adverse events was 72.5% (29/40). In the Perjeta® group, 79 TEAEs occurred in 33 subjects, and the incidence of adverse events was 82.5% (33/40). There were 132 drug-related adverse events in 158 cases. A total of 29 subjects had 71 drug-related adverse events in the SHR1309 group; the incidence of drug-related adverse reactions was 72.5% (29/40). A total of 32 subjects had 61 drug-related adverse events in the Perjeta® group; the incidence of drug-related adverse reactions was 80.0 (32/40). The main adverse reactions included hematuria, allergic dermatitis, diarrhea, rash, etc., (Table 5). In the 158 cases of TEAE, a total of 149 cases were classified as grade 1, and 9 cases were classified as grade 2. Among 132 drug-related adverse events, 126 cases were grade 1, and 6 cases were grade 2. There was no grade 3 or above adverse events in the trial (Table 5). Adverse reactions were classified according to system and organ as follows: medical examination, skin and subcutaneous tissue diseases, gastrointestinal diseases, infectious diseases, respiratory diseases, chest, and mediastinal diseases, various neurologic diseases, eye diseases, systemic diseases and reactions to drug delivery sites, heart disease, metabolic and nutritional diseases, injuries, poisoning and surgical complications, kidney and urinary system diseases. The cases of various adverse reactions are shown in Figure 3D. The incidence and severity of adverse reactions were similar between the SHR1309 and Perjeta® groups. At the end of the trial, most of the adverse reactions were on the mend. Thus, SHR1309 and Perjeta® are similar, and both have good safety.

**TABLE 5:** Summary of AEs.

AE category	SHR-1309		Perjeta®	
	Instance	Number of cases (%)	Instance	Number of cases (%)
AE category	Instance	Number of cases (%)	Instance	Number of cases (%)
TEAE	79	29 (72.5%)	79	33 (82.5%)
Drug-related AEs	71	29 (72.5%)	61	32 (80.0%)
Hematuria	16	15 (37.5)	14	13 (32.5)
Atopic dermatitis	8	7 (17.5)	11	11 (27.5)
Diarrhea	4	4 (10.0)	6	6 (15.0)
Rash	5	5 (12.5)	2	2 (5.0)
Oropharyngeal pain	1	1 (2.5)	4	4 (10.0)
Urine red blood cell positive	4	4 (10.0)	1	1 (2.5)
Grade 1	68	29 (72.5)	58	31 (77.5)
Grade 2	3	2 (5.0)	3	3 (7.5)
Above grade 2	0	0	0	0

n% is the proportion of the number of adverse reactions in all subjects who received SHR-1309 injection or Perjeta®; TEAE: treatment-emergent adverse event; drug-related AEs are defined as any AEs that are considered by the investigator to be related to the study drug.

In conclusion, compared to Perjeta® produced by Roche Pharma AG, SHR1309 produced by Shanghai Hengrui Pharmaceutical Co. Ltd. has similar pharmacokinetic, immunogenicity and safety.

## Discussion

Perjeta® is a primary drug developed by Roche Pharma AG used in the treatment of breast cancer. This experiment mainly focused on the biosimilar drug SHR1309 developed by Shanghai Hengrui Pharmaceutical Co., Ltd. The pharmacokinetics, immunogenicity and safety of SHR1309 were compared with Perjeta®. Then, the bioequivalence of SHR1309 and Perjeta® was evaluated.

Although the antibody drug SHR1309 is primarily used for female patients to treatment breast cancer, some studies have shown that Pertuzumab has a similar PK in both female patients and male subjects (Wynne et al., 2013; Kirschbrown et al., 2019). In other related studies, the subjects of Pertuzumab were also healthy male men (Kirschbrown et al., 2019). At the same time, to reduce the occurrence of ADA in women as much as possible, we chose males as subjects in this trial. According to the bioequivalence evaluation trial of some biosimilars (Wisman et al., 2014; Knight et al., 2016), and using PASS 11.0.7 to estimate the sample size, we finally determined that the number of samples in each group should be 40.

A study on SIBP-01, a biosimilar trastuzumab for HER2-overexpressing breast cancer, showed that the geometric mean ratios (90% CI) of C_max_, AUC_0-t_ and AUC_inf_ were 93.55%–104.27%, 91.98%–102.35% and 91.88–102.34% respectively, they have PK similarity (Zhou et al., 2020). And a research for a single intravenous infusion of 6 mg/kg of reference trastuzumab or its biosimilar DMB-3111 in healthy Japanese subjects showed their was bioequivalent (Morita et al., 2016). In this study, C_max_ and AUC_0-t_ were used as the main parameters for bioequivalence evaluation. Under the 90% CI, if the two compounds have similar C_max_ and AUC_0-t_ values and their geometric mean ratio is within 80.00–125.00%, we can conclude that SHR1309 is bioequivalent to Perjeta®. The results demonstrated that their mean C_max_ and AUC_0-t_ values were similar. Furthermore, the geometric ratios of most pharmacokinetic parameters were in the range of 80–125%. These findings indicate that SHR1309 and Perjeta^®^ are bioequivalent.

In another study of Unites States-trastuzumab (52 subjects) biosimilar HL02 (55 subjects), 19 subjects in both groups were ADA positive (Zhang et al., 2021). A research in healthy Chinese male volunteers, the biosimilar SIBP-01 was compared with reference product Herceptin®, ADA was detected on the 35th day after administration (Zhou et al., 2020). In this study, ADA was detected for the first time at 71st day after administration, the incidence of ADA in the two drugs was 30.0 and 32.5%, respectively. Compared to similar antibody drugs in phase I clinical trials, the positive rate of ADA was similar (Wang et al., 2020; Smolen et al., 2021). Subjects who were ADA positive were followed up for 24 months. There were no serious adverse reactions after intravenous administration of SHR1309. Most adverse reactions were grade 1, while a few were grade 2. The types of adverse reactions were also similar to Perjeta®. Compared to Perjeta®, SHR1309 exhibits similar immunogenicity and safety.

## Conclusion

In this study, a phase I clinical trial was conducted in healthy Chinese male subjects. SHR1309 can be used as a safe biosimilar of Perjeta®. This finding promotes the application of SHR1309 in clinical HER2-positive breast cancer patients.

## Data Availability

The original contributions presented in the study are included in the article/Supplementary Material, further inquiries can be directed to the corresponding author.
