# Corrigendum: Pharmacokinetics, Immunogenicity and Safety Study for SHR-1309 Injection and Perjeta^®^ in Healthy Chinese Male Volunteers

**DOI:** 10.3389/fphar.2022.907413

**Published:** 2022-05-04

**Authors:** Yingzi Cui, Dongyang Cui, Xinran Ren, Xuesong Chen, Guangwen Liu, Zhengzhi Liu, Yanli Wang, Xinyao Qu, Yicheng Zhao, Haimiao Yang

**Affiliations:** ^1^ Phase I Clinical Trial Laboratory, Affiliated Hospital of Changchun University of Chinese Medicine, Jilin, China; ^2^ Jiangsu Hengrui Medicine Co., Ltd., Lianyungang, China; ^3^ Shanghai Hengrui Pharmaceutical Co., Ltd., Shanghai, China; ^4^ School of Pharmacy, Jilin University, Jilin, China; ^5^ Clinical Medical College, Changchun University of Chinese Medicine, Jilin, China; ^6^ Jilin Province Honesty Medical Technology Consulting Co., Ltd., Jilin, China

**Keywords:** perjeta^®^, pharmacokinetics, bioequivalence, immunogenicity, safety, SHR-1309 injection

In the original article, there was a mistake in “Tables 2, 3” as published. “In Tables 2, 3, the AUC_0-t_ and AUC_0-∞_ units were set to h*ng/ml by mistake.” The corrected “Tables 2, 3” appears below.

**TABLE 2:** The main PK parameters of SHR-1309 injection or Perjeta^®^ after intravenous drip.

	SHR-1309 injection (N = 40)	Perjeta^®^ (N = 39)
C_max_ (μg/ml)	63.40 ± 15.18	64.58 ± 17.17
AUC_0-t_ (day*μg/mL)	653.37 ± 133.65	746.26 ± 197.06
AUC_0-∞_ (day*μg/mL)	657.29 ± 133.29	749.70 ± 198.23
T_max_ (h)	3.00 (0.99–48)	1.50 (1–72)
t_1/2z_ (day)	7.29 ± 2.42	7.06 ± 2.11
V_ss_ (mL/kg)	70.92 ± 11.91	66.04 ± 11.24
V_z_ (mL/kg)	49.26 ± 16.82	41.69 ± 10.94
CL_z_ (mL/h/kg)	0.20 ± 0.04	0.18 ± 0.04
λ_z_ (1/day)	0.11 ± 0.03	0.11 ± 0.03
MRT_0-t_ (day)	14.78 ± 2.08	15.61 ± 1.88
MRT_0-∞_ (day)	15.12 ± 2.01	15.87 ± 1.93
AUC_%Extrap_ (%)	0.64 ± 0.76	0.45 ± 0.46

Mean ± SD was used to describe the parameters; T_max_ was described by median (min max); C_max_, the maximum observed drug concentration in the plasma; AUC_0-t_, the AUC of the analyte in the plasma over the time interval from time zero to the last measurable concentration; AUC_0-∞_, the area under the curve from 0 to infinity; T_max_, the time from administration to the maximum observed concentration of the analyte in the plasma; t_1/2z_, the terminal half-life of the analyte in the plasma; V_ss_, the steady-state apparent distribution volume was measured after intravenous administration; V_z_, distribution volume; CL_z_, clearance rate; λ_z_, terminal rate constant in the plasma; MRT_0-t_, mean residence time from zero to the lowest detectable concentration; MRT_0-∞_, mean residence time extrapolated from zero to infinity; AUC_%Extrap_ = [(AUC_0-∞_–AUC_0-t_)/AUC_0-∞_ × 100].

**TABLE 3:** Results of the equivalence determination of the test drug and reference drug.

PK parameter	Geometric mean		Comparison	
	SHR-1309 injection (N = 40)	Perjeta^®^ (N = 39)	Ratio%	90% CI (%)
C_max_ (μg/ml)	61.92	63.53	97.47	89.66–105.9
AUC_0-t_ (day*μg/mL)	639.65	734.07	87.14	80.07–94.83
AUC_0-∞_ (day*μg/mL)	643.88	737.43	87.31	80.27–94.98
t_1/2z_ (day)	6.97	6.84	101.81	90.62–114.39
V_ss_ (mL/kg)	69.83	64.47	108.33	101.19–115.96
V_z_ (mL/kg)	46.86	40.17	116.67	103.93–130.96
CL_z_ (mL/h/kg)	0.19	0.17	114.59	105.30–124.69
MRT_0-t_ (day)	14.61	15.58	93.77	89.13–98.65
MRT_0-∞_ (day)	14.61	15.84	94.54	90.04–99.26

PK, pharmacokinetic; CI, confidence interval; C_max_, maximum observed drug concentration in the plasma; AUC_0-t_, the AUC of the analyte in the plasma over the time interval from time zero to the last measurable concentration; AUC_0-∞_, the area under the curve from 0 to infinity; t_1/2z_, the terminal half-life of the analyte in the plasma; V_ss_, steady-state apparent distribution volume was measured after intravenous administration; V_z_, distribution volume; CL_z_, clearance rate; MRT_0-t_, mean residence time from zero to the lowest detectable concentration; MRT_0-∞_, mean residence time extrapolated from zero to infinity.

In the original article, there was an error. “The AUC_0-t_ and AUC_0-∞_ units were set to h*ng/ml by mistake.”

A correction has been made to “Results,” “Pharmacokinetics,”:

“To evaluate the bioequivalence of SHR-1309 and Perjeta^®^, we performed PK analysis on two groups of subjects. The subjects were sampled at 21 time points before and after drug administration. Plasma drug concentration was detected by ELISA, and the data were fitted to form the average plasma drug concentration curve of SHR-1309 and Perjeta^®^ (**Figure 2A**). The logarithmic transformation of the curve is shown in **Figure 2B**. At the same time, the plasma drug concentration of each subject in the two groups was fitted (**Figures 2C,D**). There was no significant difference in blood concentration between the two groups after administration. The primary evaluation parameters, secondary evaluation parameters and other pharmacokinetic parameters were obtained through calculation of plasma drug concentration (Table 2; **Supplementary Table S1**). The mean and standard deviation (SD) values of C_max_ were 63.40 ± 15.18 μg/ml and 64.58 ± 17.17 μg/ml for SHR-1309 and Perjeta^®^, respectively, and the ratio of the geometric mean was 98.30%. The mean and SD values of AUC_0-t_ were 653.37 ± 133.65 and 746.26 ± 197.06 day*μg/mL, respectively, and the ratio of the geometric mean was 88.41%. The mean and SD values of AUC_0-∞_ were 657.29 ± 133.29 and 749.70 ± 198.23 day*μg/mL, respectively, and the ratio of the geometric mean was 88.58%. T_max_ was 1.50 and 3.00 h, respectively. The geometric mean values and ratios of all parameters are shown in Table 3. The primary pharmacokinetic parameters of SHR-1309 and Perjeta^®^ were all up to the standard. Except for Vz, the 90% CI for all values fell within the 80%–125% range (**Figure 3A**). The PK parameter values of the two drugs were similar, and according to the PK evaluation standard of bioequivalence, SHR-1309 and Perjeta^®^ are bioequivalent.”

The authors apologize for this error and state that this does not change the scientific conclusions of the article in any way. The original article has been updated.

