# Supplementary material for: Pharmacokinetics, Immunogenicity and Safety Study for SHR-1309 Injection and Perjeta® in Healthy Chinese Male Volunteers
Source: Front Pharmacol. 2021 Jun 2;12:660541. doi: 10.3389/fphar.2021.660541 (PMC8207516; doi:10.3389/fphar.2021.660541)
Supplement: Supplementary file 1 [file DataSheet1.docx]

**Supplementary information**

Phase I clinical study on the pharmacokinetics, immunogenicity and safety of SHR-1309 injection and Perjeta^®^ in healthy Chinese male volunteers

Yingzi Cui, Dongyang Cui, Xinran Ren, Guangwen Liu, Zhengzhi Liu, Yanli Wang, Xinyao Qu, Yicheng Zhao, Haimiao Yang

**Inclusion criteria and exclusion criteria**

**Inclusion criteria** (Volunteers should meet all the inclusion criteria before they can be selected)

1. The subjects voluntarily sign the informed consent approved by the ethics committee, and fully understand the trial content, process and possible adverse reactions;

2. The subjects should be able to complete the study according to the requirements of the trial protocol;

3. The subjects are willing to take effective contraception measures within 6 months after signing the informed consent to drug administration;

4.The subjects are limited to healthy males, aged 18-55 years old (including 18 and 55 years old);

5. Body weight was no less than 50 kg, body mass index (BMI) between 19–26 kg / m^2^ (Including critical value); body mass index (BMI) = weight (kg) / height ^2^ (m^2^);

6. The left ventricular ejection fraction (LVEF) was within the normal range (> 50%) by echocardiography before randomization;

7. The skin at the injection site is intact without tattoo or lesion;

8. According to the results of all physical examinations, vital signs, electrocardiograms, chest radiographs and prescribed clinical laboratory examinations, all of them are normal or abnormal but without clinical significance.

**Exclusion criteria** (Volunteers were excluded if they met one of the exclusion criteria)

1. Subjects who are known to be allergic to Patuzumab or its analogues or injection excipients, or subjects with allergic constitution (two or more kinds of drugs and food);

2. The subjects who have difficulty in venous blood collection or could not tolerate venipuncture;

3. The subjects who have undergone major surgery in the first 3 months or planned surgery during the study period;

4. The subjects who had or are suffering from any clinically important diseases such as motor system, circulatory system, endocrine system, nervous system, digestive system, respiratory system, hematology, immunology, psychiatry or metabolic abnormality, and were judged by the research doctors not suitable for participating in this trial;

5. Smokers or those who smoke more than 5 cigarettes per day in 3 months before the trial;

6. Alcoholics or regular drinkers within 6 months before screening, those who drink more than 14 units of alcohol per week on average (1 unit = 360 ml beer or 45 ml 40% alcohol or 150 ml wine), or who drank alcoholic beverages within 48 hours before administration of the study drug;

7. Those who lost blood or donated more than 400 ml blood within 3 months before the trial;

8. Currently suffering from heart disease, including but not limited to chronic heart failure (CHF), hypertension or hypotension, the subjects with abnormal blood pressure at the time of screening and admission (systolic blood pressure < 90 mmHg or > 140 mmHg, and / or diastolic blood pressure < 50 mmHg or > 90 mmHg);

9. Subjects with a history of postural hypotension, syncope or vertigo and shock of any cause;

10. The subjects with chronic or acute infectious diseases and clinical significance in screening period and admission;

11. The subjects who have previously received the treatment of Patuzumab or her dimerization inhibitor, or other antibody or protein drugs targeting HER-2 receptor;

12. Those who received live vaccine 30 days before screening or need to be vaccinated during the screening period to the end of the study;

13. Those who had used monoclonal antibody or biological preparation for injection or biological drug injection within one year before using the research drug;

14. Those who participated in the drug clinical trial within 3 months before the trial;

15. Those who plan to receive biological preparations for injection or biological drug injection within one year after the administration of the study drug;

16. HBsAg, HCV antibody, treponema pallidum antibody or HIV positive subjects;

17. Subjects with acute disease during the screening or before study;

18. The subjects who took part in strenuous exercise 96 hours before the experiment or took part in strenuous sports, including physical contact exercise or impact exercise, within 30 days after the drug administration;

19. Alcohol screening positive subjects

20. Those with a history of drug abuse or positive drug screening;

21. Those who have special requirements for diet and cannot abide by the unified diet;

22. The researchers judged that there are a low possibility of enrollment (including inability to understand the research requirements, poor compliance, weakness, etc.) or that there were other factors that were not suitable to participate in the study.

Supplementary Table 1. The main PK parameters of the SHR-1309 injection or Perjeta^®^ after intravenous drip.

|  | SHR-1309 injection (N = 40) | Perjeta^®^ (N = 39) |
| --- | --- | --- |
| C_max_ (ng/mL)  Mean ± SD  CV%  Min-Max  Median  Q1-Q3 | 63.40 ± 15.18  23.9  37.1-111  59.90  54.25-69.10 | 64.58 ± 17.17  26.6  39.9-145  61.15  55.20-70.80 |
| AUC_0-t_ (h*ng/mL)  Mean ± SD  CV%  Min-Max  Median  Q1-Q3 | 653.37 ± 133.65  20.5  398.89-954.92  646.67  583.43-732.15 | 746.26 ± 197.06  26.4  421.97-1364.20  701.85  604.15-829.45 |
| AUC_0-∞_ (h*ng/mL)  Mean ± SD  CV%  Min-Max  Median  Q1-Q3 | 657.29 ± 133.29  20.3  407.23-956.12  648.22  585.55-733.92 | 749.70 ± 198.23  26.4  422.90-1369.01  716.33  605.44-830.47 |
| T_max_ (h)  Mean ± SD  CV%  Min-Max  Median  Q1-Q3 | 3.36 ± 7.32  217.7  0.99-48  3.00  1.25-3.00 | 3.80±11.12  292.5  1-72  1.50  1.00-3.00 |
| t_1/2z_ (day)  Mean ± SD  CV%  Min-Max  Median  Q1-Q3 | 7.29 ± 2.42  33.2  3.90-13.06  6.62  5.48-9.27 | 7.06 ± 2.11  29.9  3.97-12.11  6.64  5.57-8.37 |
| V_ss_(mL/kg)  Mean ± SD  CV%  Min-Max  Median  Q1-Q3 | 70.92 ± 11.91  16.8  43.09-96.23  72.02  62.18-80.92 | 66.04 ± 11.24  17.0  35.09-90.05  67.34  60.15-72.03 |
| V_z_(mL/kg)  Mean ± SD  CV%  Min-Max  Median  Q1-Q3 | 49.26 ± 16.82  34.2  23.05-90.61  42.93  37.22-64.57 | 41.69 ± 10.94  26.2  24.62-62.86  39.51  33.53-48.04 |
| CL_z_(mL/h/kg)  Mean ± SD  CV%  Min-Max  Median  Q1-Q3 | 0.20 ± 0.04  21.5  0.13-0.31  0.19  0.17-0.21 | 0.18 ± 0.04  24.0  0.09-0.29  0.17  0.15-0.21 |
| λ_z_ (1/ day)  Mean ± SD  CV%  Min-Max  Median  Q1-Q3 | 0.11 ± 0.03  31.5  0.05-0.18  0.10  0.07-0.13 | 0.11 ± 0.03  28.0  0.06-0.17  0.10  0.08-0.12 |
| MRT_0-t_(day)  Mean ± SD  CV%  Min-Max  Median  Q1-Q3 | 14.78 ± 2.08  14.1  10.47-18.72  14.89  13.46-16.29 | 15.61 ± 1.88  12.1  12.67-19.65  15.91  13.95-16.95 |
| MRT_0-∞_(day)  Mean ± SD  CV%  Min-Max  Median  Q1-Q3 | 15.12 ± 2.01  13.3  10.92-19.46  15.41  13.60-16.51 | 15.87 ± 1.93  12.2  12.78-19.94  16.12  14.07-17.17 |
| AUC_%Extrap_ (%)  Mean ± SD  CV%  Min-Max  Median  Q1-Q3 | 0.64 ± 0.76  119.0  0.11-3.26  0.31  0.16-0.69 | 0.45 ± 0.46  103.2  0.09-2.41  0.26  0.16-0.54 |

C_max_: The maximum observed drug concentration in the plasma; AUC_0-t_: the AUC of the analyte in the plasma over the time interval from time zero to the last measurable concentration; AUC_0-∞_: the area under the curve from 0 to infinity; T_max_: the time from administration to the maximum observed concentration of the analyte in the plasma; t_1/2z_: the terminal half-life of the analyte in the plasma; V_ss_: The steady-state apparent distribution volume was measured after intravenous administration; V_z_: distribution volume; CL_z_: clearance rate; λ_z_: terminal rate constant in the plasma; MRT_0-t_: Mean residence time from zero to the lowest detectable concentration; MRT_0-∞_: Mean residence time extrapolated from zero to infinity; AUC_%Extrap_ =[(AUC_0-∞_-AUC_0-t_)/AUC_0-∞_] ×100%; Mean ± SD was used to describe the parameters; T_max_ was described by median (min max); CV%: Coefficient of Variation. Min: minimum; Max: maximum; Q1-Q3: First quartile -Third quartile.
